# Tenofovir-associated renal dysfunction in clinical practice: An observational cohort from western India

**DOI:** 10.4103/2589-0557.68998

**Published:** 2010

**Authors:** Ketan K. Patel, Atul K. Patel, Rajiv R. Ranjan, Apurva R. Patel, Jagdish K. Patel

**Affiliations:** Infectious Diseases Consultant, Infectious Diseases Clinic, Navarangpura, Ahmedabad - 380 009, India; 1 Adit Molecular Diagnostic Center, “VEDANTA” Institute of Medical Sciences, Navarangpura, Ahmedabad - 380 009, India

**Keywords:** Antiretroviral therapy, nephrotoxicity, renal dysfunction, tenofovir

## Abstract

**Background:**

Tenofovir (TDF) is preferred nucleoside reverse transcriptase inhibitors (NRTI) for the treatment of human immunodeficiency virus infection because of its potency and safety. Renal toxicity with TDF use is low and comparable with other NRTI in clinical trials, but there are many case studies and small case series of renal dysfunction with TDF.

**Materials and Methods:**

This is an observational longitudinal cohort of patients started on a TDF-based regimen from January 2007 to April 2010. Patients were evaluated at baseline and with every follow-up visit for serum creatinine and calculated creatinine clearance (Cockroft-Gault formula). In addition to this, the patients were also subjected to test for serum potassium, phosphorous and urine examinations as and when indicated. Renal dysfunction was defined as rise in serum creatinine to more than the upper level of normal (>1.2 mg%).

**Results:**

Of 1,271 patients started on a TDF-containing antiretroviral treatment (ART) 83 (6.53%) developed renal dysfunction, of which 79 had impaired serum creatinine and five had Fanconi’s syndrome. Renal dysfunction was more common with boosted a protease inhibitor (PI) (9.44%)-based regimen as compared to a non- nucleoside reverse transcriptase inhibitors (NNRTI) (5.01%)-based regimen (*P* = 0.003). The mean decline in creatinine clearance from baseline was 22.27 ml/min. The median time to develop renal dysfunction was 154 (15–935) days. Serum creatinine returned to normal in all the patients after stopping TDF. Five patients presented with features suggestive of Fanconi’s syndrome without alteration in serum creatinine.

**Conclusion:**

TDF-based treatment is associated with mild but reversible renal dysfunction. Patients receiving PI/r are at a higher risk of renal dysfunction compared to those receiving NNRTI-based ART. Clinicians should be adviced to have intensive renal monitoring, including creatinine clearance, urine examination, K+ and phosphate levels at baseline and during treatment with TDF.

## INTRODUCTION

Tenofovir disoproxil fumarate (TDF) is an oral prodrug of tenofovir, an acyclic nucleoside phosphonate. TDF is the first nucleotide analogue reverse transcriptase inhibitor to be approved for the treatment of human immunodeficiency virus (HIV) infection.[1–4]

TDF is extensively excreted by the renal route by means of glomerular filtration, with 20–30% being actively transported into the renal proximal tubule cells by organic anion transporter 1 (OAT-1). Boosted protease inhibitor (PI/r)-based therapies can increase the plasma exposure of TDF by 20–30%.[5]

TDF is generally considered safe; however, renal toxicity has been reported with its use.[5–9] Even though TDF most often has been reported to cause proximal renal tubulopathy, e.g., Fanconi syndrome, other related nephrotoxicities, including diabetes insipidus, calcium and phosphorus dysregulation with bone disease,[10] and reduction in glomerular function have also been reported.[11,12] Although the incidence of nephrotoxicity appears to be low (ranging from 0.3 to 2%), many caregivers will see this complication develop due to the agent’s widespread use. Risk factors include low body weight, impaired renal function at baseline and concomitant receipt of nephrotoxic drugs.[13–15] Although in most cases tubular dysfunction is reversible after withdrawal of TDF,[4,9] persistent renal damage with renal dysfunction has been reported.[13,16] Here, we describe renal dysfunction in patients started on a TDF-containing regimen.

## MATERIALS AND METHODS

In this observational longitudinal cohort, patients started on a TDF-based regimen were evaluated at baseline and with every follow-up visit for serum creatinine and calculated creatinine clearance. In addition to this, the patients were also subjected to test for serum potassium, phosphorous and urine examinations as and when indicated. Baseline demographic data were collected. Renal dysfunction was defined as[1] rise in serum creatinine to more than the upper level of normal (>1.2 mg%).[2] Laboratory evidence of Fanconi syndrome, e.g. hypokalemia, hypophosphatemia and normoglycemic glycosuria. Creatinine clearance was calculated using the Cockroft–Gault formula. Data were analyzed using descriptive statistics and chi-square test.

## RESULTS

**Figure 1:**
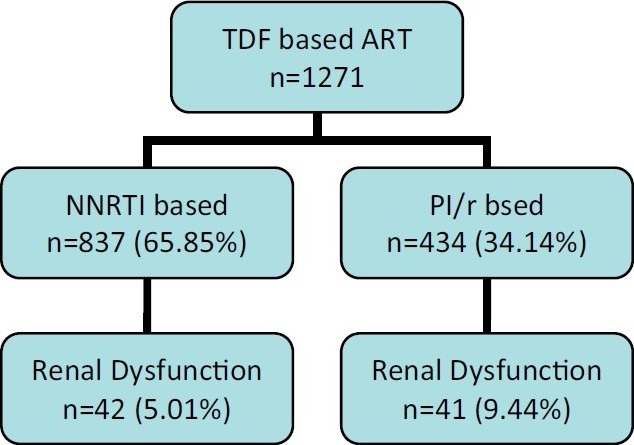
Flow chart of patient started on a TDF-based ART

From January 2007 to April 2010, 1,271 patients were started on a TDF-containing regimen. Details are given in Figure 1.

In the NNRTI group, 784 and 53 patients were on efavirenz (EFV) and nevirapine (NVP)-based regimens, respectively, while in the boosted PI group, 188, 132, 103 and 11 patients were on atazanavir/r (ATV/r), indinavir/r (IDV/r), lopinavir/r (LPV/r) and saquinavir/r (SAQ/r)-based regimens, respectively. The baseline characteristics of patients are as given in Table 1.

**Table 1:** Baseline characteristics of patients

Parameters	Total (*n* = 83)
Rise in creatinine	Number (*n* = 78)
Age in years, median (range)	44 (30–72); 46.52 (10.94)
Sex	
Male	63
Female	15
Weight in kilograms, median (range)	60 (40–112)
Time to develop renal dysfunction in days, median (range)	154 (15–935)
Creatinine at baseline in mg%, median (range)	1 (0.7–1.2); 0.99 (0.12)
Creatinine clearance at baseline in mL/min, median (range)	76.55 (35.24–182.11)
Repeat creatinine in mg%, median (range)	1.38 (1.21–3.69)
Repeat creatinine clearance in mL/ min, median (range)	54.28 (23.10–105.91)
Fanconi syndrome	*n* = 5
Male	2
Female	3

Of all the patients (*n* = 1,271), a total of 83 (6.53%) developed TDF renal dysfunction, of which 42 (5.01%) patients were started on an NNRTI-based regimen, while 41 (9.44%) were on a PI/r-based regimen. Of the 83 patients, 78 (6.14%) patients had developed rise in creatinine level to more than the upper limit of normal and five patients (0.39%) had developed proximal tubular dysfunction. Median time to developing renal dysfunction was 154 (15–935) days.

**Figure 2:**
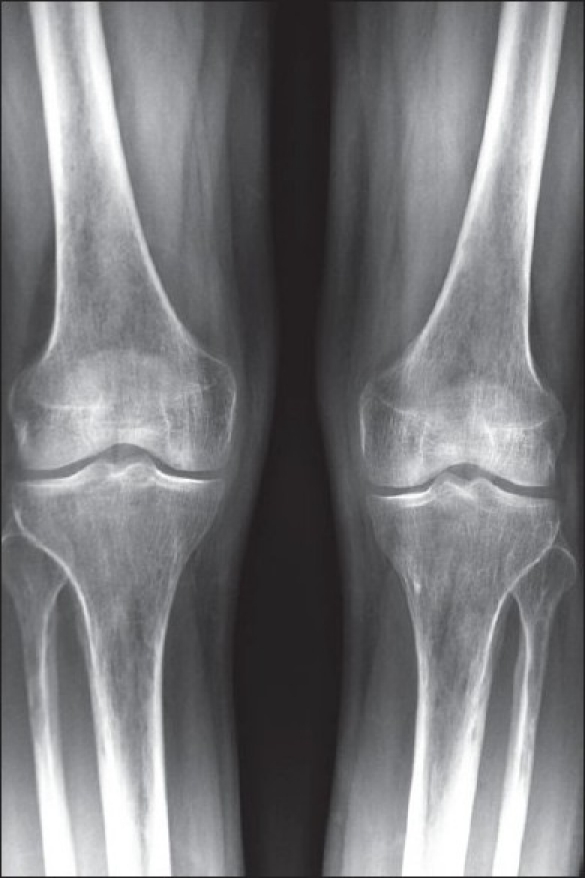
X-ray of both knees showing osteoporosis

Mean decline in creatinine clearance from baseline was 22.27 mL/min at the time of renal dysfunction. Urine examination of these patients with elevated serum creatinine showed either normal examination or trace to 1+ albumin without active sediments. All patients with impaired serum creatinine recovered after discontinuation of TDF. Three patients with elevated serum creatinine who were allowed to continue TDF for further 2 weeks showed a further worsening of the serum creatinine. This suggests a drug related renal dysfunction most likely a tubulo-interstitial injury. Patients with rise in serum creatinine were largely asymptomatic while five patients presented with weakness, bone pains and backache; of these, two had severe pain and had difficulty in walking. All five patients had refractory hypokalemia and hypophosphatemia with normal serum creatinine levels, and two of them had normoglycemic glycosuria to complete features of Fanconi syndrome. Two patients who underwent Dual energy x-ray absorptiometry (DEXA) scans showed severe osteoporosis. Plain X-ray examination of both knees in patient with bone pains showed osteoporosis [Figure 2].

Patients on the PI/r-based regimen had higher chances of developing TDF toxicity compared to patients on the NNRTI-based regimen (*P* = 0.003). Ritonavir dosage/day (200 mg vs. 100 mg) was not found to be associated with an increased risk of TDF renal dysfunction (*P* = 0.1582). Similarly, no statistically significant difference was observed for TDF-associated renal dysfunction between the ATV/r (100 mg of ritonavir/day) and NNRTI group (*P* = 0.4212). The observed renal dysfunction with different regimens used was as follows: EFV = 39 (4.97%), NVP = 2 (3.77%), SAQ = 2 (18.18%), ATV = 13 (6.91%), IDV = 13 (9.85%), LPV = 9 (8.74%).

The associated comorbidities and possible risk factors for renal dysfunction in our cohort were hypertension/CAD (12.04%), chronic liver diseases (HCV, 9.63%), prolonged survival with HIV (>10 years, 8.43%), diabetes (8.43%), renal calculi (7.23%) and single kidney (1.20%).

No patients received concomitant nephrotoxic drugs.

## DISCUSSION

TDF is currently recommended as a first-line agent in combination with other antiretrovirals for HIV management because of its favorable pharmacokinetic profile, good antiviral potency, high tolerability and low incidence of mitochondrial toxicities. Because of the availability of generic medicine and efficacy-safety profile, TDF usage has increased in developing countries. TDF has been considered safe and associated with fewer side-effects, including renal dysfunction, in clinical trials.[1–4] However, there have been many case reports and cohort studies describing TDF-associated nephrotoxicity.[5–9,13,17–20]

Clinically, the spectrum of TDF-associated nephrotoxicity spans all levels of severity, from mild renal tubular dysfunction with subclinical decline in renal function to classical Fanconi syndrome.

The majority of the patients (6.14%) in our cohort had elevated creatinine with normal urine examination, except trace to 1+ albuminuria, which suggests tubulointerstitial injury due to TDF, while 0.39% had clinical and biochemical features favoring proximal tubular dysfunction. Patients in our cohort who were allowed to continue TDF showed progressive renal injury. which favors drug-related injury.

Known risk factors for the development of TDF-induced nephrotoxicity include underlying renal dysfunction, low CD4 count and low body weight,[13,14,16,21] concomitant use of boosted PI and other nephrotoxic agents. Of the 164 patients with TDF-induced Fanconi syndrome reported to the FDA, the majority (83%) were treated with protease inhibitors, specifically, 74% had received a ritonavir-boosted regimen. Although discontinuation of TDF results in renal recovery in the majority of the cases, some patients experience chronic kidney disease. Similar to our observation (p=0.003, other studies have found that patients receiving ritonavir-boosted PI have greater TDF-associated renal function decline compared to the NNRTI-based therapy,[22,23] In all these patients, laboratory abnormalities rapidly improved after stopping the TDF. The median body weight in our cohort was 60 (40–112) kg, suggesting that lesserweight may not be an important factor predisposing to renal dysfunction. Other risk factors for renal dysfunction in our cohort were hypertension/CAD (12.04%), chronic liver diseases (HCV-related, 9.63%), prolonged survival with HIV (>10 years, 8.43%), diabetes (8.43%), renal calculi (7.23%) and single kidney (1.20%). Rachel *et al*., in his study, showed that antiretroviral-naive patients have a statistically significant raised creatinine levels with an increasing probability of developing raised creatinine level during follow-up (*P* < 0.001), which contrast with our finding. This study also brings out other factorsth, such as non-compliance, hepatitis C virus coinfection and intravenous drug use, as risk factors for TDF toxicity.[21]

The mechanisms underlying these renal toxicities are not fully understood. TDF and its more nephrotoxic sister drugs, adefovir and cidofovir, undergo active tubular secretion. Because intracellular drug accumulation is a function of uptake and secretion, enhanced uptake via the OAT-1 on the basolateral membrane or impaired efflux via one or more of the apical transporters can, in theory, result in drug accumulation and potential toxicity.[7,13] Mitochondrial DNA depletion by the accumulated high intracellular drug levels has been proposed as a mechanism of the renal toxicity associated with NRTI use.[24,25] It is important to note that TDF is a weak inhibitor of DNA polymerase gamma than are the other NRTIs. Administration of ritonavir alone or with lopinavir has been shown to increase the maximum serum concentrations of tenofovir by >30%, and it is likely that ritonavir increased the proximal tubular concentrations of tenofovir by decreasing its urinary secretion, thereby leading to TDF toxicity.

In our cohort, higher ritonavir dosage (200 vs. 100 mg/day) as a PI booster was not associated with an increased incidence of renal dysfunction with TDF.

The article by Irizarry–Alvarado and coworkers highlights not only the synergistic nephrotoxic potential of TDF and didanosine,[19] fortunately a rarely used NRTI combination in current practice, but also points out the need to monitor renal function, phosphate levels, urinalysis for glycosuria and urinary protein excretion on a regular basis in patients receiving TDF.

As suggested before,[26] we also recommend especially for clinicians practicing in resource-limited settings to be vigilant and keep looking for TDF-related renal toxicity, which although reversible can lead to significant morbidity.

Through a very simple definition used to define renal dysfunction in this manuscript, it gives important information to sensitize the practicing doctors about TDF-associated renal toxicity. As described, majority of the patients had milder and reversible renal dysfunction following TDF use. This information should be useful for a clinician to diagnose TDF related renal toxicity early and manage its severe forms with associated morbidity.

## CONCLUSIONS

TDF-based treatment is associated with mild but reversible renal dysfunction. Patients receiving PI/r are at a higher risk of renal dysfunction compared to NNRTI-based ART. Clinicians are adviced to monitor renal function aggressively by calculating creatinine clearance and doing a urine examination, K ^+^ and phosphate levels at baseline and during follow up with TDF use.
